# On the Optimization of Regression-Based Spectral Reconstruction †

**DOI:** 10.3390/s21165586

**Published:** 2021-08-19

**Authors:** Yi-Tun Lin, Graham D. Finlayson

**Affiliations:** School of Computing Sciences, University of East Anglia, Norwich NR4 7TJ, UK; G.Finlayson@uea.ac.uk

**Keywords:** spectral reconstruction, hyperspectral imaging, multispectral imaging, regression, regularization, inverse problem

## Abstract

Spectral reconstruction (SR) algorithms attempt to recover hyperspectral information from RGB camera responses. Recently, the most common metric for evaluating the performance of SR algorithms is the Mean Relative Absolute Error (MRAE)—an $\ell_{1}$ relative error (also known as percentage error). Unsurprisingly, the leading algorithms based on Deep Neural Networks (DNN) are trained and tested using the MRAE metric. In contrast, the much simpler regression-based methods (which actually can work tolerably well) are trained to optimize a generic Root Mean Square Error (RMSE) and then tested in MRAE. Another issue with the regression methods is—because in SR the linear systems are large and ill-posed—that they are necessarily solved using regularization. However, hitherto the regularization has been applied at a spectrum level, whereas in MRAE the errors are measured per wavelength (i.e., per spectral channel) and then averaged. The two aims of this paper are, first, to reformulate the simple regressions so that they minimize a relative error metric in training—we formulate both $\ell_{2}$ and $\ell_{1}$ relative error variants where the latter is MRAE—and, second, we adopt a per-channel regularization strategy. Together, our modifications to how the regressions are formulated and solved leads to up to a 14% increment in mean performance and up to 17% in worst-case performance (measured with MRAE). Importantly, our best result narrows the gap between the regression approaches and the leading DNN model to around 8% in mean accuracy.

## 1. Introduction

A consumer RGB camera captures light signals with three types of color sensors. Yet the light is physical radiance—a continuous spectral function of wavelength—which, intuitively, can hardly be described by a 3-dimensional color representation. Indeed, many researchers have found that real-world spectra should be at least 5- to 8-dimensional [1,2,3,4,5]. Consequently, with RGB imaging we can only acquire limited information encoded in the light spectrum.

Using a hyperspectral camera [6,7,8,9,10,11,12,13,14,15,16,17,18,19], we can record scene radiance at high spectral and spatial resolution. This technique has been widely used in machine vision applications such as remote sensing [20,21,22,23,24,25,26,27], medical imaging [28,29,30,31], food processing [32,33,34,35,36,37], and anomaly detection [38,39,40,41,42,43,44], as well as in the spectral characterization domain, including the calibration of color devices (e.g., cameras [45] and scanners [46]), scene relighting [47,48], and art conservation and archiving [49,50,51]. While useful, hyperspectral cameras are usually much more expensive than the RGB cameras. Moreover, the extra spectral information is often captured with reduced spatial and/or temporal resolution, which limits their usefulness.

Spectral reconstruction (SR) seeks to recover spectral information from the RGB data of a single camera  [52,53,54,55,56,57,58,59,60,61,62,63,64,65,66]. Assuming the recovery error of SR is low enough (regarding the results in the literature and in this paper), we can essentially measure spectra using an RGB camera.

Historically, SR is most efficiently solved by Linear Regression (LR) [52], where the map from RGBs to spectra is described by a simple linear transformation. Considering a nonlinear map, the Polynomial Regression (PR) [53] and Root-Polynomial Regression (RPR) [56] methods expand the RGBs into a set of polynomial/root-polynomial terms—which are then mapped to spectra via a linear transform. More recent regression models, including the Radial Basis Function Network (RBFN) [54] and the A+ sparse coding algorithm [55], use clustering techniques to define the local neighborhood (in the color or spectral space) in which each RGB is regressed.

On the other hand, we see most of the recent SR algorithms are not based on simple regressions but based on Deep Neural Networks (DNN) [60,61,62,63,64,65,66] which embrace the idea of regressing RGB image patches as a whole (as oppose to regressing each pixel independently in regressions). This approach hypothesizes that object-level descriptions of the RGBs can—though requiring much more computational resources—aid the recovery of spectra. However, DNN-based models do not always perform better than simple regressions [55] and often suffer from instability issue when recovering spectra at different brightness scales [56,57,58]. Furthermore, spectra recovered by DNNs are shown to be less accurate in color [57,59] and do not necessarily provide more accurate cross-illumination or cross-device color reproductions [57].

In this paper, we aim to further optimize the existing regression-based SR methods. First, we noticed that in recent works, DNN-based models are evaluated and ranked by Mean Relative Absolute Error (MRAE) [63,64]. Most top DNN models are also designed to minimize MRAE directly [60,61,62,63,64]. However, all regressions used in SR are optimized by convention for Root Mean Square Error (RMSE) while evaluated using MRAE (or other similar relative errors) when compared with the DNNs [55,56,57,60,61]. In other words, these regressions are optimized for one metric, and then how well they work is evaluated with another.

**Figure 1:**
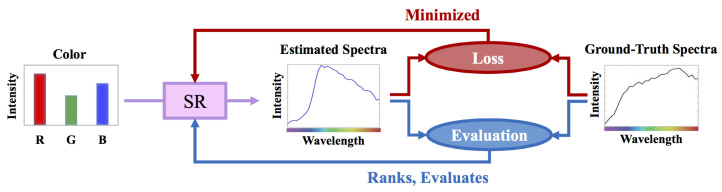
The standard spectral reconstruction (SR) training and evaluation scheme.

In Figure 1, we illustrate the standard experimental framework of SR. In training, the parameters of the SR model are tuned such that the “losses”—the differences between the ground-truths and estimations measured by a given loss metric—are statistically minimized. After the SR models are trained, we evaluate them based on a desired evaluation metric. Ideally, the loss and evaluation metrics should match (i.e., the same or similar in nature). Indeed, a model that is optimized for one metric but evaluated by another will surely lead to sub-optimal results.

Based on this insight, we propose two new minimization approaches for simple regressions—the Relative Error Least Squares (RELS) and Relative Error Least Absolute Deviation (RELAD). While the former minimizes an error similar to MRAE and is solved in closed form, the latter explicitly minimizes the MRAE metric but has the disadvantage of requiring an iterative minimization.

The second contribution of this paper is to propose a new way of regularizing the regression-based spectral reconstruction. Most regressions are necessarily trained using a regularization constraint [67], both to prevent overfitting and to make the system equations more “stable” [68] (a system of equations is stable if small perturbations appear in the training data results in a small perturbation in the solved-for model parameters). However, we observe that hitherto in regression-based spectral reconstruction all spectral channels are regularized altogether—e.g., in [52,53,54,55,56]. That is the regularization constraint is effectively applied at the spectrum level. Yet, fundamentally, error metrics such as MRAE measure the errors at all spectral channels independently and then average them to give a spectral error measure. Thus, we propose a “per-channel” regularization methodology which ensures optimized regularization for each spectral channel independently.

**Figure 2:**
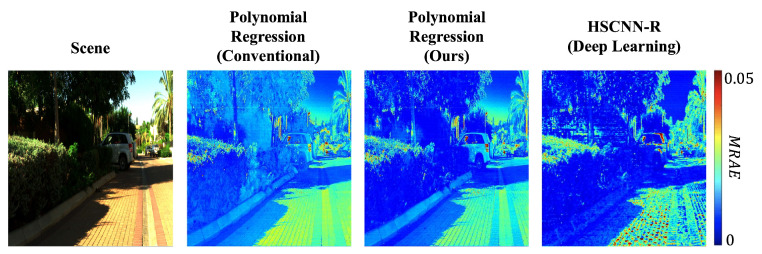
Example hyperspectral image reconstruction error heat maps (in MRAE) by the conventional Polynomial Regression [53] (**left**), Polynomial Regression based on our new RELS method (**middle**), and the deep learning-based HSCNN-R [60] (**right**).

Combined, we find that training the simple regressions to minimize the same error as used in testing and adopting a per-channel regularization approach lead to a significant uptick in performance. Moreover, as shown in the example hyperspectral image reconstruction results in Figure 2, our new regression methods deliver performance that is similar to one of the best (but much more complex) DNN approaches.

The rest of the paper is organized as follows. In Section 2, we will introduce the prerequisites of regression-based spectral reconstruction. Our new methods are introduced in Section 3. Section 4 details the experimental procedures, and Section 5 discusses the experimental results. This paper concludes in Section 6.

## 2. Background

### 2.1. Regression-Based Spectral Reconstruction

A simple mathematical model of RGB formation is written as [69]

(1) $$\underline{x}=\int_{\Omega }r\left(\lambda \right)\underline{s}\left(\lambda \right)\;d\lambda \;,$$

where r(λ) denotes the radiance spectrum, $\underline{s}\left(\lambda \right)={\left[s_{R}\left(\lambda \right),s_{G}\left(\lambda \right),s_{B}\left(\lambda \right)\right]}^{T}$ is the set of three spectral sensitivities of the color sensors, and $\underline{x}={\left[R\;G\;B\right]}^{T}$ is the derived color vector (the superscript ${}^{T}$ denotes the transpose operator). In the case of color imaging, the range of integration, Ω, is the visible range. In this paper, we consider this range to be the interval from 400 to 700 nanometers (nm).

Further, we assume r(λ) and $\underline{s}\left(\lambda \right)$ are measured by a hyperspectral device at every 10 nm (in accordance with the recent SR challenge datasets [63,64]). As such, we can approximate the integration in Equation (1) by inner products [69]:

(2) $$\underline{x}=S^{T}\underline{r}\;,$$

where $\underline{r}={\left[r\left(400\right),r\left(410\right),\cdots ,r\left(700\right)\right]}^{T}$ is the 31-component vectorized spectrum, and S is the 31×3 spectral sensitivity matrix (one spectral sensitivity per column).

In SR, we study the inverse problem: we wish to recover $\underline{r}$ using the information of $\underline{x}$. A general definition of SR (considering both regressions and DNNs) can be written as

(3) $$\Psi \left(N_{k}\right)\approx {\underline{r}}_{k}\;,\;N_{k}=\left\{{\underline{x}}_{k,1},{\underline{x}}_{k,2},\cdots ,{\underline{x}}_{k},\cdots ,{\underline{x}}_{k,n}\right\}\;,$$

where ${\underline{r}}_{k}$ denotes the hyperspectral data at the location of interest (the kth pixel), Ψ denotes an SR algorithm, and the neighborhood set $N_{k}$ includes the RGBs in the spatial neighborhood of pixel *k* (the subscripts ${}_{k,l}$ indexing the lth pixel in the proximity of pixel *k*). Note that $N_{k}$ also contains the RGB at the location of interest i.e., ${\underline{x}}_{k}$.

In regression we normally do not use the neighborhood information to recover spectra (though small neighborhoods are occasionally admitted [55,56]). Rather, we attempt to map each RGB uniquely to a corresponding spectrum, i.e., $\Psi \left({\underline{x}}_{k}\right)\approx {\underline{r}}_{k}$.

#### 2.1.1. Linear Regression

The simplest Linear Regression (LR) approach [52] models the SR algorithm Ψ by a linear transformation:

(4) $$\Psi \left({\underline{x}}_{k}\right)=M^{T}{\underline{x}}_{k}\approx {\underline{r}}_{k}\;,$$

where $M^{T}$ is a 31×3 matrix.

Considering the whole database of *N* spectra and their matching RGBs (regardless of which images they are in and their pixel locations), let us arrange them into the N×31 spectral data matrix $R={\left[{\underline{r}}_{1},{\underline{r}}_{2},\cdots ,{\underline{r}}_{k},\cdots ,{\underline{r}}_{N}\right]}^{T}$ and the matching RGB data matrix $X={\left[{\underline{x}}_{1},{\underline{x}}_{2},\cdots ,{\underline{x}}_{k},\cdots ,{\underline{x}}_{N}\right]}^{T}$. Based on this nomenclature, we can rewrite Equation (4) as

(5) XM≈R.

That is, all spectra (the rows of R) are estimated from their corresponding RGBs (the rows of X) using the same linear transformation matrix M.

#### 2.1.2. Nonlinear Regressions

The regression problem summarized in Equation (5) is, by construction, linear. In a general case where we may wish to pose the SR problem as a non-linear regression, we might transform each row of X (each RGB) to an *s*-component counterpart using a given nonlinear function $\varphi :R^{3}\mapsto R^{s}$:

(6) $$X_{\varphi }={\left[\varphi \left({\underline{x}}_{1}\right),\varphi \left({\underline{x}}_{2}\right),\cdots ,\varphi \left({\underline{x}}_{k}\right),\cdots ,\varphi \left({\underline{x}}_{N}\right)\right]}^{T}\;.$$

This N×s transformed RGB matrix is then regressed to estimate the spectral data R using a new linear transformation matrix $M_{\varphi }$:

(7) $$X_{\varphi }M_{\varphi }\approx R\;.$$

Here, the dimension of $M_{\varphi }$ is s×31.

Examples of nonlinear regressions in the literature include Polynomial Regression (PR) [53], Root-Polynomial Regression (RPR) [56], and Radial Basis Function Network (RBFN) [54]. For instance, if we consider the 2nd-order RPR, we will have $\varphi \left(\underline{x}\right)={\left[R\;G\;B\;\sqrt{RG}\;\sqrt{GB}\;\sqrt{BR}\right]}^{T}$, in which case $X_{\varphi }$ is an N×6 matrix and the regression matrix $M_{\varphi }$ is 6×31 [56].

#### 2.1.3. A+ Sparse Coding

So far, both linear and nonlinear regressions assume a single regression matrix that is used to estimate spectra for all RGBs. Yet in A+ sparse coding (i.e., adjusted anchored neighborhood regression) [55], it is considered that the SR mapping should be done locally [55,59]. Fundamentally, in sparse coding one assumes that all spectra can be represented by linear combinations of a small number of anchor spectra (hence it is “sparse”) [55,70].

In training, the A+ algorithm runs the K-SVD clustering algorithm [71] and assigns the clusters’ centers as the set of anchor spectra. Around the corresponding RGB value of each anchor, a fixed number of nearest RGB neighbors in the training dataset and their corresponding radiance spectra are recorded. These neighboring data points are then used to create a bespoke Linear Regression mapping (Equation (5)) that should be only used by the RGBs in the testing set that are around the same neighborhood [59]. That is, in the reconstruction stage of A+, we first find the anchor spectrum whose matching RGB is closest to the query RGB, and second we map the query RGB to spectrum using the regression mapping associated with that neighborhood.

We note that all the discussed regression models can be formulated by either Equations (5) or (7), while the structure of both are essentially the same. Thus, to simplify the notation we will adopt the regression notation of Equation (5), i.e., we drop the subscript ${}_{\varphi }$ of Equation (7). As such, in later discussions the data we are regressing can be the RGBs or their non-linear expansions thereof.

### 2.2. Least-Squares Minimization

Now let us consider how we solve for the regression matrix M. In essence, we must define what we meant by the ≈ symbol in Equation (5).

Most commonly, we seek to minimize the “sum-of-squares” between the estimations and ground-truths:

(8) $$\min_{M}\;\;||XM-R|{|}_{2}^{2}\;.$$

Here, $\left|\right|\cdot {\left|\right|}_{2}^{2}$ calculates the sum of all components squared. Equivalently, $\left|\right|\cdot {\left|\right|}_{2}^{2}$ is sometimes written as $\left|\right|\cdot {\left|\right|}_{F}^{2}$ denoting the Frobenius norm.

Regressions solved in this manner are called the Least-Squares (LS) regression. Advantageously, the solution of LS minimization can be solved in closed form [72].

#### 2.2.1. The Overfitting Problem and Regularized Least Squares

However, the solution of Equation (8) often cannot be directly used in practice—given that the solved regression matrix M is optimized for minimizing the errors in the training set, we often find that this matrix does not work for unseen data. In other words, denoting a set of unseen RGB and spectral data as $X^{\prime }$ and $R^{\prime }$, we often get $X^{\prime }M≉R^{\prime }$. This problem is so-called “overfitting” [67,68].

One of the biggest problems for an overfitted regression is that it might not work in the presence of noise. As a thought experiment, we may consider to recover R (the same set of training spectral data) from the “perturbed” training RGB data, $X^{\prime }=X+\epsilon$, where ϵ is a matrix of very small numbers, representing the noise occurs in the RGB imaging process. It follows that an overfitted M can very possibly suggest $X^{\prime }M=\left[X+\epsilon \right]M≉R$, that is, it fails to plausibly recover R.

Another facet of this problem is that if we actually attempt to find the best regression matrix for the noisy data [X+ϵ], we can end up having the optimal regression matrix that is very different from the original one. That is, as we perturb our RGB data, we can arrive at very different regressions.

To mitigate the overfitting problem, the tool of ridge regularization (a.k.a. Tikhonov regularization) [67] is often incorporated when training a regression. While solving the minimization in Equation (8), we add another penalty term which bounds the magnitude of the regression matrix M:

(9) $$\min_{M}\;||XM-R|{|}_{2}^{2}+\gamma ||M|{|}_{2}^{2}\;,$$

where the solution of M can still be written in closed form [52].

Here, the γ parameter—which is set by the user—controls how ${\left||M|\right|}_{2}^{2}$ mitigates the minimization of the sum-of-squares fitting error. Clearly, if γ=0, the equation resorts to the simple LS (Equation (8)), and as γ becomes large, the need to solve for an M that has a small (bounded) norm becomes imperative and the requirement to reduce the sum-of-squares fitting error is less important.

When the solved M has a bounded norm, the regression has the stability property we desire. That is, if we perturb X by a small amount we will still get the same (or very similar) M, and this in turn implies that albeit the perturbation in the input RGBs we will still get similar spectral estimations. We refer the readers to the work in [67] for a fuller discussion of how regularization is used and why it solves the instability problem.

**Figure 3:**
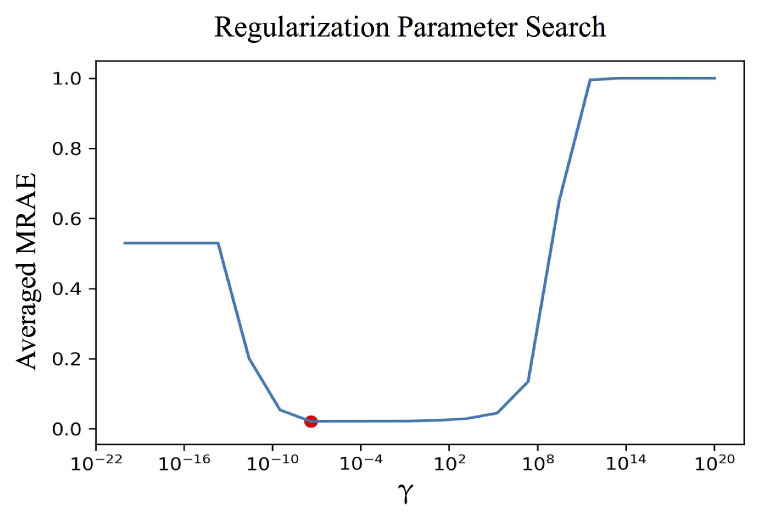
An illustration of searching for the regularization parameter. The averaged MRAE (vertical axis) is calculated over the validation set (a separate data set that is not the training set). The red dot in the graph indicates the minimal error and the suggested regularization parameter.

In practice, we choose the γ parameter empirically to best trade-off the need for a stable solution and to lower the fitting spectral errors. Figure 3 illustrates a typical cross-validation parameter selection methodology [73]. First, a wide range of different γ’s are tried, and the solved M’s depending on these γ values are used to recover spectra in an unseen “validation set”, which are then evaluated using the desired evaluation metric (here, we use the MRAE metric). Usually, a “U-shaped” curve is obtained when plotting the averaged validation-set MRAE against γ, where the minimal point (the red dot in the plot) indicates the selected parameter.

We note that the regularization parameter (the red dot in Figure 3) is not “fixed”, that is we search for independent regularization parameter in every minimization instance encountered.

While the ridge regularization approach introduced here (adding an $\ell_{2}$ penalty term) is the most widely used approach (not only for SR but for learning problems in general), there are other regularization approaches used in the literature, including the LASSO [74] (where the $\ell_{1}$ penalty term is used) and Elastic Net approach [75] (where both $\ell_{1}$ and $\ell_{2}$ penalty terms are added, with dependent or independent regularization parameters). It is known that LASSO provides more robust regularization compared to the $\ell_{2}$ ridge regularization, while the Elastic Net provides the possibility to swing more towards LASSO or ridge regression depending on the relative dominance between the two methods in each specific learning circumstances. For more information of these two methods, interested readers are pointed to the works in [74,75], respectively.

### 2.3. RMSE versus MRAE Error Measures

In the prior art of SR, most (if not all) regression-based methods are based on LS minimization with a ridge regularization setting (see, e.g., in [52,53,54,55,56]). As the LS minimization uses sum-of-squares as its loss metric (see Equations (8) and (9)), a natural metric for evaluating LS-based regressions is Root Mean Square Error (RMSE):

(10) $$\mathrm{RMSE}\left(\hat{\underline{r}},\underline{r}\right)=\sqrt{\frac{1}{31}||\hat{\underline{r}}-\underline{r}|{|}_{2}^{2}}\;$$

(because the sum-of-squares is essentially the sum of squared RMSE). Here, we denote $\hat{\underline{r}}=\Psi \left(\underline{x}\right)$ as the estimation (returned by, e.g., regressions or DNNs) of the ground-truth $\underline{r}$.

Of course, the physical radiance spectra may have greater or lesser magnitudes depending on the imaging conditions [56]. However, if we model the change in brightness by a scalar *k*, we get $\mathrm{RMSE}\left(k\hat{\underline{r}},k\underline{r}\right)=k\mathrm{RMSE}\left(\hat{\underline{r}},\underline{r}\right)$. Which means, for a ground-truth spectrum that is *k* times brighter than $\underline{r}$, when compared with the recovered spectrum also scaled by *k* (i.e., $k\hat{\underline{r}}$), we will get *k* times larger RMSE than the original case.

This biased nature of RMSE evaluation can as well influence the training process of regressions. Indeed, we can expect that the standard LS minimization can overestimate the squared-RMSE (i.e., sum-of-squares) losses of the bright spectra and consequently place more importance on minimizing their errors compared to the dim ones.

This brightness bias is part of the motivation underpinning the Mean Relative Absolute Error (MRAE) metric for spectral recovery [63,64]. This MRAE metric almost universally adopted in the recent literature is defined as

(11) $$\mathrm{MRAE}\left(\hat{\underline{r}},\underline{r}\right)=\frac{1}{31}||\frac{\hat{\underline{r}}-\underline{r}}{\underline{r}}|{|}_{1}\;,$$

where the spectral difference is divided by (component-wise) the ground-truth spectral values. As such, the effect of brightness level is discounted when measuring spectral differences, i.e., $\mathrm{MRAE}\left(\hat{k\underline{r}},k\underline{r}\right)=\mathrm{MRAE}\left(\hat{\underline{r}},\underline{r}\right)$.

Arguably, the MRAE makes more sense as a performance metric as the SR algorithm might be used where the absolute/maximum intensity of the tested spectra is not known or controlled. Therefore, the main goal of this paper is to consider how to modify the SR regression to minimize a relative recovery error (in general and MRAE in particular).

We would also like to emphasize that the development of our new methods (presented in the following sections) are, of course, tailored to the assumption that MRAE is used as the evaluation error metric. Moreover, again, MRAE is certainly the most commonly used metric adopted for DNNs in the literature at this time, see, e.g., in [60,61,63,64].

## 3. Rethinking the Optimization of Regression for Spectral Reconstruction

The first part of our contributions concerns the regularization method used in the standard case (Equation (9)), where the regularization takes place at the spectrum level with all spectral channels being regularized together. Here, we will argue—and develop the requisite mathematics—that the regularization should be done per spectral channel.

In our second contribution, we alter the form of the regression. Following that recently MRAE is used to evaluate and rank new methods, and that this metric is optimized directly in modern DNN solutions but not in regression-based methods, we reformulate the regressions to optimize for fitting errors that are “relative” in nature (spectral differences relative to the ground-truth values). We develop solutions based on both $\ell_{2}$ and $\ell_{1}$ relative error minimizations.

### 3.1. Per-Channel Regularization

Returning to the conventional regression formulation in Equation (5), let us split the regression matrix M and the ground-truth spectral data R by columns:

(12) $$X\left[{\underline{m}}_{1},{\underline{m}}_{2},\cdots ,{\underline{m}}_{i},\cdots ,{\underline{m}}_{31}\right]\approx \left[{\underline{\rho }}_{1},{\underline{\rho }}_{2},\cdots ,{\underline{\rho }}_{i},\cdots ,{\underline{\rho }}_{31}\right]\;.$$

Here, ${\underline{\rho }}_{i}$ denotes the ith column of R. Remember that each row of R is a single radiance spectrum, thus the numbers in ${\underline{\rho }}_{i}$ are the ith-channel spectral intensities of all the spectra in the database.

Now, we observe that the original multivariate regression is in fact a collection of 31 independent regressions:

(13) $$X{\underline{m}}_{i}\approx {\underline{\rho }}_{i}\;\mathrm{for}\;i=1,2,\cdots ,31.$$

Notice that Equation (13) is equivalent to the original formulation in Equation (5), only that it explicitly shows there is no “inter-channel” dependence. In other words, for all regression-based SR in the literature, it has been always the case that each column of M is only used for recovering the corresponding column of R while irrelevant to the recoveries for other spectral channels.

Curiously, as we solve for M using the standard LS minimization in Equation (9), the strength of the penalty term, ${\gamma ||M||}_{2}^{2}$, is controlled by a single parameter γ, meaning that all columns of M, that is, the 31 ${\underline{m}}_{i}$’s, are regularized using the same γ parameter despite the fact that each of them works independently of others. Essentially, by regularizing M as a whole, we are “asserting” such an interdependence among channels.

From a mathematical viewpoint (regarding how the regression is formulated), it makes more sense to regularize each per-channel regression independently. Following Equation (13), we rewrite Equation (9) in a per-channel fashion:

(14) $$\min_{{\underline{m}}_{i}}\;||X{\underline{m}}_{i}-{\underline{\rho }}_{i}|{|}_{2}^{2}+\gamma_{i}||{\underline{m}}_{i}|{|}_{2}^{2}\;\mathrm{for}\;i=1,2,\cdots ,31\;.$$

Here, the per-channel regularization parameter $\gamma_{i}$ (again to be optimized by a cross-validation procedure, see Section 2.2.1) is used specifically for regularizing the regression of the ith spectral channel. That is, we select different regularization parameters for different spectral channels.

We would like to remark that, although our per-channel approach matches the assumption made by the regression’s formulation (that there is no inter-spectral-channel dependence), we shall admit the possibility that there might be better ways to formulate the regression which factors in “reasonable interdependence” between channels. For example, we may consider to impose a “smoothness” constraint used in the literature (see, e.g., in [76]) on the recovered spectra, though we note that this assumption would be more important for the recovery of “reflectance” spectra (which are intrinsically smooth) instead of the “radiance” spectra we are recovering (because the illumination spectrum is part of the radiance, which can occasionally make the radiance far from smooth, especially for indoor illuminations).

The solution of Equation (14) can be written in closed form [67]:

(15) $${\underline{m}}_{i}={\left[X^{T}X+\gamma_{i}I\right]}^{-1}X^{T}{\underline{\rho }}_{i}\;.$$

Here, the I matrix is the identity matrix—if Linear Regression (Equation (5)) is used, then I is 3×3; otherwise, if a nonlinear transform is incorporated (Equation (7)), I is s×s where *s* is the dimension of the nonlinear feature vectors.

### 3.2. Relative-Error Least Squares Minimization

Now, let us minimize an error that is more similar to MRAE. First, we consider to formulate an $\ell_{2}$-minimization problem so as to ensure a closed-form solution.

From Equation (5), we can remodel the approximation as the following minimization:

(16) $$\min_{M}\;||\frac{XM-R}{R}|{|}_{2}^{2}\;=\;\min_{{\hat{\underline{r}}}_{1},{\hat{\underline{r}}}_{2},\cdots ,{\hat{\underline{r}}}_{N}}\;\sum_{k=1}^{N}||\frac{{\hat{\underline{r}}}_{k}-{\underline{r}}_{k}}{{\underline{r}}_{k}}|{|}_{2}^{2}\;\;,\;{\hat{\underline{r}}}_{k}=M^{T}{\underline{x}}_{k}\;,$$

where all the divisions are component-wise. Here, the square of an $\ell_{2}$ relative error (referred to as the “relative-RMSE” in some works [55,70]) is minimized. We call this new minimization approach the Relative Error Least Squares (RELS).

Equation (16) can be rewritten in a per-channel fashion:

(17) $$\min_{{\hat{\underline{\rho }}}_{1},{\hat{\underline{\rho }}}_{2},\cdots ,{\hat{\underline{\rho }}}_{31}}\;\sum_{i=1}^{31}||\frac{{\hat{\underline{\rho }}}_{i}-{\underline{\rho }}_{i}}{{\underline{\rho }}_{i}}|{|}_{2}^{2}\;\;,\;{\hat{\underline{\rho }}}_{i}=X{\underline{m}}_{i}\;$$

(these two minimizations are equivalent because the divisions are component-wise). Or equivalently, we write

(18) $$\min_{{\underline{m}}_{i}}\;||\frac{X{\underline{m}}_{i}}{{\underline{\rho }}_{i}}-\underline{1}|{|}_{2}^{2}\;\mathrm{for}\;i=1,2,\cdots ,31\;,$$

where $\underline{1}$ is an *N*-component vector of ones.

We can further define a matrix of RGBs weighted by the reciprocals of ${\underline{\rho }}_{i}$:

(19) $$H_{i}=\frac{X}{{\underline{\rho }}_{i}}=\left(\begin{array}{cccc} 1/\rho_{i,1} & 0 & \cdots & 0\\ 0 & 1/\rho_{i,2} & \cdots & 0\\ \vdots & \vdots & \ddots & \vdots \\ 0 & 0 & \cdots & 1/\rho_{i,N} \end{array}\right)X\;,$$

where ${\underline{\rho }}_{i}={\left[\rho_{i,1},\rho_{i,2},\cdots ,\rho_{i,N}\right]}^{T}$. Using this nomenclature, let us again rewrite Equation (18) into

(20) $$\min_{{\underline{m}}_{i}}\;||H_{i}{\underline{m}}_{i}-\underline{1}|{|}_{2}^{2}\;\mathrm{for}\;i=1,2,\cdots ,31\;.$$

Clearly, Equation (20) shows that RELS is in effect another least-squares problem which regresses $H_{i}$ to fit the vector $\underline{1}$.

Of course, we need to regularize this minimization by solving the following equation instead:

(21) $$\min_{{\underline{m}}_{i}}\;||H_{i}{\underline{m}}_{i}-\underline{1}|{|}_{2}^{2}+\gamma_{i}||{\underline{m}}_{i}|{|}_{2}^{2}\;\mathrm{for}\;i=1,2,\cdots ,31\;,$$

whose closed-form solution is written as [67,77]

(22) $${\underline{m}}_{i}={\left[H_{i}^{T}H_{i}+\gamma_{i}I\right]}^{-1}H_{i}^{T}\underline{1}\;.$$

### 3.3. Relative-Error Least Absolute Deviation Minimization

Finally, let us consider to minimize MRAE directly. Analogous to Equation (16), we are now going to solve the following minimization:

(23) $$\min_{M}\;||\frac{XM-R}{R}|{|}_{1}=\min_{{\hat{\underline{r}}}_{1},{\hat{\underline{r}}}_{2},\cdots ,{\hat{\underline{r}}}_{N}}\;\sum_{k=1}^{N}||\frac{{\hat{\underline{r}}}_{k}-{\underline{r}}_{k}}{{\underline{r}}_{k}}|{|}_{1}\;,\;{\hat{\underline{r}}}_{k}=M^{T}{\underline{x}}_{k}\;.$$

Following the same derivation as Equations (16)–(21), we reach

(24) $$\min_{{\underline{m}}_{i}}\;||H_{i}{\underline{m}}_{i}-\underline{1}|{|}_{1}+\gamma_{i}||{\underline{m}}_{i}|{|}_{1}\;\mathrm{for}\;i=1,2,\cdots ,31\;.$$

In the literature, regressions solved via an $\ell_{1}$ minimization is called the Least Absolute Deviation (LAD) [78,79]. As here the MRAE we are minimizing is a relative error, we call this new approach the Relative Error Least Absolute Deviation (RELAD).

Notice that for the regularization penalty term in Equation (24) we also adopted an $\ell_{1}$ norm, which refers to the LASSO regularization [74] (see Section 2.2.1).

Unlike RELS, RELAD does not have a closed-form solution. For a small amount of data, a globally optimal solution can be found using a linear-programming solution [78,80]. However, in our application the amount of data is large—where the Iterative Reweighted Least Squares (IRLS) [79] algorithm is more appropriate and is thus used here.

The IRLS process approaches RELAD minimization by repeatedly solving Weighted Least Squares (WLS) [81] while updating the weights on every iteration depending on the losses and mapping functions obtained in the previous iteration, until the solution converges.

The detailed algorithm is given in Algorithm 1. The iteration number is indicated by the superscript ${}^{\left(t\right)}$. All min, division, and absolute operations shown in Algorithm 1 are component-wise to the vectors, while the median function in Step 8 and the mean functions in Step 11 calculate the median and mean of components of ${\underline{\delta }}^{\left(t\right)}$ (the resulting scalar $\hat{\sigma }$ in Step 8 is a preliminary estimate of the standard deviation of absolute losses commonly used in the literature, see, e.g., in [79,82]). Furthermore, the min functions used in Step 9 and 10 are to clip the reciprocal values at $10^{6}$ so as to prevent overly large numbers. Finally, in Step 11, we set the tolerance ϵ=0.00005 and the stopping iteration T=20.
**Algorithm 1:** Solving RELAD regression (Equation (24)) by IRLS algorithm.1:$W^{\left(0\right)}={\tilde{W}}^{\left(0\right)}=I$       ▷ Initialization of weights; I is the N×N identity matrix2:${\underline{\delta }}^{\left(0\right)}=\inf$             ▷ Absolute losses of all data are initialized to infinity3:t=04:**repeat**5:    t=t+16:    ${\underline{m}}_{i}^{\left(t\right)}={\left[H_{i}^{T}W^{\left(t-1\right)}H_{i}+\gamma_{i}{\tilde{W}}^{\left(t-1\right)}\right]}^{-1}H_{i}^{T}W^{\left(t-1\right)}\underline{1}$   ▷ Closed-form WLS solution7:    ${\underline{\delta }}^{\left(t\right)}=|H_{i}{\underline{m}}_{i}^{\left(t\right)}-\underline{1}|$                      ▷ Absolute losses8:    $\hat{\sigma }=\frac{\mathrm{median}\left({\underline{\delta }}^{\left(t\right)}\right)}{0.6745}$        ▷ Preliminary estimate of the standard deviation of ${\underline{\delta }}^{\left(t\right)}$9:    $W^{\left(t\right)}=\hat{\sigma }\times diag\left(\min\left(\frac{1}{{\underline{\delta }}^{\left(t\right)}},\;10^{6}\right)\right)$      ▷ The × operator is the scalar product10:    ${\tilde{W}}^{\left(t\right)}=diag\left(\min\left(\frac{1}{\left|{\underline{m}}_{i}^{\left(t\right)}\right|},\;10^{6}\right)\right)$11:**until**$|\mathrm{mean}\left({\underline{\delta }}^{\left(t\right)}\right)-\mathrm{mean}\left({\underline{\delta }}^{\left(t-1\right)}\right)|<\epsilon$**or**t≥T12:${\underline{m}}_{i}={\underline{m}}_{i}^{\left(t\right)}$                         ▷ Return the converged ${\underline{m}}_{i}$

## 4. Experiment

### 4.1. The Regression Models

The regression models we consider in this paper (introduced in Section 2.1) are listed in Table 1. For each of these regressions, we train the model under the 4 minimization criteria listed in Table 2. That is, we benchmark 5 models paired with 4 minimization approaches; but, irrespective of which minimization we are using, we evaluate the performance of all algorithms using MRAE.

For reference, we benchmark against HSCNN-R [60]—the 2nd place winner of the NTIRE 2018 challenge [63]. HSCNN-R is DNN-based (orders of magnitude more parameters compared to all considered regressions), and it minimizes MRAE directly.

All implemented codes are provided as the Supplementary Materials.

### 4.2. Preparing Ground-Truth Datasets

In this paper, we adopt the ICVL hyperspectral image database [70], which was the database used for the NTIRE 2018 Spectral Reconstruction Challenge [63]. Each spectral image from the database (200 in total) has a spatial dimension of 1392×1300—that is, approximately 1.8 M spectra per image—with 31 spectral channels (referring to 10-nm spectral samplings between 400 and 700 nm).

For each spectral image, we calculate the corresponding RGB image following Equation (2), using the CIE 1964 color matching functions [83,84] as the camera’s spectral sensitivities S, i.e., the resulting RGBs are the CIEXYZ tristimulus values [84]. This setting corresponds to the “Clean Track” methodology of NTIRE 2018 and 2020 Spectral Reconstruction Challenge [63,64].

### 4.3. Training, Validation, and Testing

We follow a 4-trial cross-validation process. First, we randomly separated all images (pairs of hyperspectral and corresponding RGB images) into 4 data subsets—denoted as image subset A, B, C, and D; each consists 50 scenes. Then, in each trial, 2 subsets were used for training, 1 subset was used for validation, and 1 subset was used for testing, and the roles for each subset in different trials were permuted as follows:

- Trial #1—Training: A & B, Validation: C, Testing: D
- Trial #2—Training: A & B, Validation: D, Testing: C
- Trial #3—Training: C & D, Validation: A, Testing: B
- Trial #4—Training: C & D, Validation: B, Testing: A.

In the training process of each trial, the training set was used to minimize for either the LS, LS${}^{\mathrm{pc}}$, RELS, or RELAD criteria.

Then, the validation set was used to determine the unknown regularization parameters following the procedure introduced in Section 2.2.1. More specifically, we first coarsely tried a wide range of magnitudes: γ or $\gamma_{i}=\left\{10^{-20},10^{-19},\cdots ,10^{20}\right\}$, and then selected the magnitude that returns the lowest averaged validation-set MRAE. Next, we conducted a finer search at 1000 points (equidistant on the log scale) between the two adjacent magnitudes of the coarsely selected one, which determines the final selection of γ or $\gamma_{i}$.

For the deep learning-based HSCNN-R, the validation step is different. In HSCNN-R, we use the validation set to determine the termination epoch of the training [60]. The actual termination epochs adopted differ among the 4 cross-validation trials, but all of them are between 315 and 350 epochs.

Finally, we evaluated all models and minimization criteria using the testing-set images. The resulting statistics presented in the next section are the averaged testing results over the 4 cross-validation trials.

## 5. Results

### 5.1. Mean and Worst-Case Performance

In Table 3, we present the mean statistics of per-image-mean and per-image-99-percentile (worst-case) MRAE over the 200 tested images. Let us first look at the numbers in the first row, which are results of variations of LR (i.e., Linear Regression). The mean results (under the headline “Mean per-image-mean MRAE”) show that LR trained using all of our three new approaches outperform the standard LS (least-squares), among which the RELAD method performs the best—returning 14% lower MRAE compared to LS. On the right side of Table 3 (headlined “Mean per-image-99-percentile MRAE”), we see that RELS-based LR provides 17% lower worst-case MRAE compared to using the standard LS. Likewise, observing all other regression models, we found that the best minimization criterion in terms of mean MRAE is either RELS or RELAD depending on the model.

To further examine the robustness of the best minimization approach against other tested approaches, we present the “paired” (or “dependent”) two-sample Student’s *t*-test scores [85] in Table 4. The *t*-test scores are calculated based on the observed 200 pairs of per-image-mean MRAEs delivered by two compared minimization criteria. For example, let us look at the top-left number of the table, where 9.46 is the *t*-test score when comparing the “Best” approach for LR (i.e., RELAD, which delivers the lowest mean per-image-mean MRAE for LR) versus the conventional LS minimization. In the same row, we also see the *t*-test scores comparing the Best approach with LS${}^{\mathrm{pc}}$ and RELS, respectively, but not RELAD (because RELAD is the Best criterion itself).

In essence, the larger the absolute value of the *t*-test score, the more significant the two distributions are apart (i.e., the advantageous mean performance of the Best criterion has statistical significance). In our case, all tests have a degree of freedom of 200−1=199 (200 is the number of dependent sample pairs). Accordingly, with a “one-sided” hypothesis (as we only want to test if the mean of the Best criterion is smaller), a 5% level of significance (so-called the “*p*-value” in most literature) corresponds to a *t*-score of 1.65 (according to the *t*-distribution table in [86]). Evidently, we see that the *t*-scores of all our tests are greater than the 1.65 threshold. In a practical sense, this result suggests that for each regression model, using the corresponding best minimization criterion can consistently deliver the lowest per-image-mean MRAEs of all.

Still, it is somewhat counterintuitive that RELAD performs worse than RELS in some cases. Indeed, as RELAD directly minimizes MRAE, we might expect that RELAD would always provide the lowest mean MRAE. This outcome likely originates from the imprecision problem of IRLS specifically when solving LAD (i.e., the $\ell_{1}$ Least Absolute Deviation) minimizations [79,87,88]. Another possible cause which might result in suboptimal optimization is that the IRLS algorithm might, in some cases, fail to converge after 20 iterations (remember that we set the stopping iteration T=20 in Step 11 of Algorithm 1). We stopped iterating after 20 iterations to keep limit the computational complexity of the problem.

In contrast, it is not entirely surprising that RELS has better worst-case performance than RELAD (and consequently RELS is the best method for all tested regressions in terms of the worst-case MRAE). Indeed, it is well known that $\ell_{1}$ minimizations tend to be less sensitive to outliers compared to their $\ell_{2}$ counterparts [78,79]. Which means, in RELAD these 99-percentile pixels might be treated as outliers during training—i.e., minimization of these pixels are less important—and thus perform worse in testing.

Finally, it is shown that our best performing regression model—the RELS-based PR—is only 8.7% worse than HSCNN-R in terms of mean MRAE. We remind the reader that HSCNN-R is one of the top models in the NTIRE 2018 challenge [63] in which all finalists are based on DNNs. Given that most of the reported challenge entries were much more than 10% worse than HSCNN-R under the MRAE evaluation, we can expect that some of our further optimized regression models can be on par with—or even better than—many DNN models in the challenge.

This result contradicts the assumption taken by many DNN approaches that mapping large image patches is necessary for achieving top performances—as all tested regressions in our experiment are pixel-based. Furthermore, regarding how much fewer model parameters the regressions use in comparison to the DNNs (e.g., the PR regression model has 2573 parameters, whereas HSCNN-R has approximately $10^{7}$ parameters), it is surprising to see that regression methods can achieve comparable performance to the DNNs (let alone being better than some). As DNNs have millions of parameters, it is likely that the training data is insufficient to robustly optimize these parameters (the ICVL database [70] used in our study and in NTIRE 2018 challenge [63] is already one of the largest available hyperspectral image databases so far).

### 5.2. Computational Time

The training and reconstruction time of all tested models are presented in Table 5. We calculate the training time as the average time used for each cross-validation trial, and the reconstruction time as the average time used to reconstruct each tested image.

Our hardware specification includes Intel${}^{®}$ Core${}^{\mathrm{TM}}$ i7-9700 CPU and NVIDIA${}^{®}$ GeForce${}^{®}$ RTX 2080 SUPER${}^{\mathrm{TM}}$ GPU. Note that the GPU is only used to run the training process of the HSCNN-R model. All testing (reconstruction) processes and the training of the regressions use solely the CPU.

Let us look at the training time (the left side of Table 5). First, we see that regardless of the regression model, the closed-form LS, LS${}^{\mathrm{pc}}$ and RELS approaches show absolute dominance over the iteratively solved RELAD approach and HSCNN-R for shorter training time. Then, although it appears that the RELAD minimization in general takes longer than training HSCNN-R, we remark that HSCNN-R is GPU-accelerated (which is highly based on parallel programming), yet our implementation of RELAD does not use any apparent parallel programming technique.

As for the reconstruction time results (the right side of Table 5), we see that all tested regression methods require much less time to reconstruct a spectral image compared to HSCNN-R. Additionally, we notice that the reconstruction time for regression models does not depend on the adopted minimization approach—this result makes sense because after all, no matter how we optimized the regression matrix, the reconstruction procedure is the same. Consequently, in the case that longer “offline” training time is permitted, the RELAD-optimized regression can still support fast reconstruction as LS, LS${}^{\mathrm{pc}}$, and RELS.

### 5.3. Brightness Dependence

**Figure 4:**
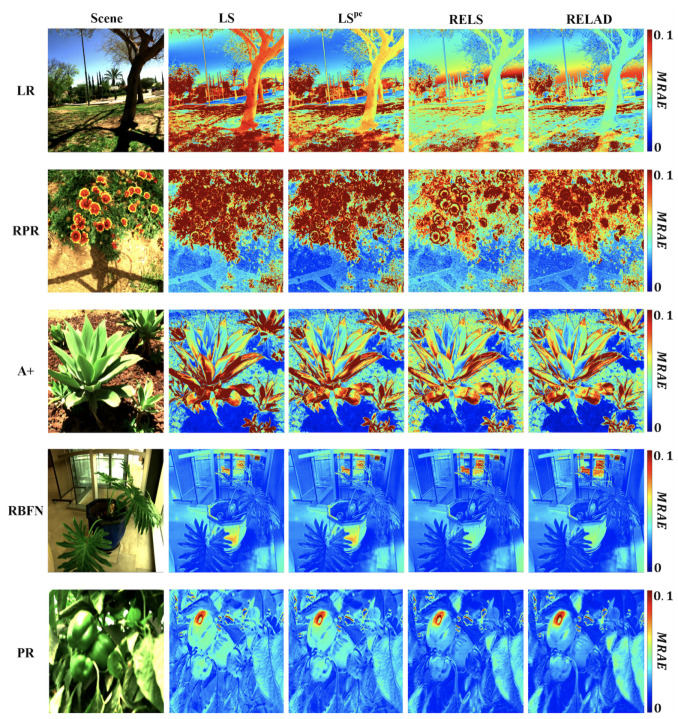
The MRAE error heat maps for the considered regression models optimized for LS, LS${}^{\mathrm{pc}}$, RELS, and RELAD criteria.

Let us further investigate the performance discrepancy within an image. In Figure 4, we show for each regression approach the MRAE recovery error map of a given example scene. We see that RELS and RELAD tend to improve the spectral recoveries for the foreground objects particularly e.g., trees and grass in the LR results, flowers in the RPR results, leaves in the A+ results, bonsai pot in the RBFN results, and the green peppers in the PR results. Yet, it seems that in the background and/or highlight regions (e.g., sandy grounds and the blue sky), LS and LS${}^{\mathrm{pc}}$ in turn outperform RELS and RELAD.

We observe that generally in scenes included in the ICVL database the foregrounds are dimmer than the backgrounds, therefore we presume the reason that LS and LS${}^{\mathrm{pc}}$ tend to recover spectra of the background and highlights more accurately (yet perform much worse in the foreground) is due to the brightness bias of least-squares minimization (see Section 2.3).

**Figure 5:**
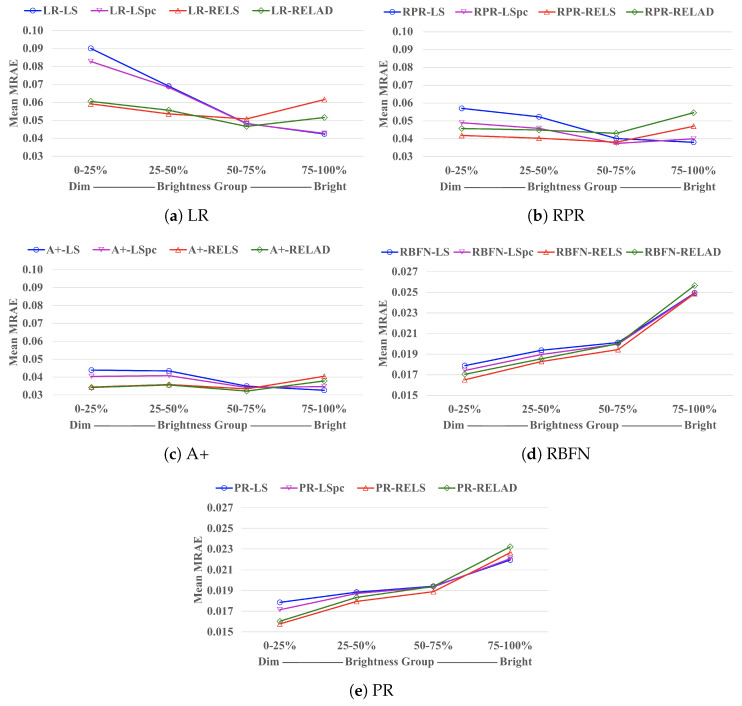
The mean MRAE performance for pixels belonging to 4 different percentile groups (based on their brightness) in each image. For display purposes, in plot (**a**–**c**) the Mean MRAE (the vertical axes) is shown between the interval of [0.03,0.10], while in plot (**d**,**e**) it is shown between [0.015,0.027].

In Figure 5, we exhibit how the mean MRAE performance of each regression approach is related to the pixels’ brightness. Here, “brightness” is defined as the $\ell_{2}$ norm of the ground-truth spectrum. For each image we separated all pixels into 4 different brightness groups—0–25 percentile (the dimmest group), 25–50 percentile, 50–75 percentile, and 75–100 percentile (the brightest group). Then, for pixels belonging to the same brightness group (from all testing-set images) we calculated their mean MRAE, which is presented in the plots.

Clearly, we observe that while LS and LS${}^{\mathrm{pc}}$ generally provide lower MRAE for the brightest group in an image (75–100%), RELS and RELAD demonstrate great advantages over LS and LS${}^{\mathrm{pc}}$ in the two dimmest groups (0–25% and 25–50%), which eventually leads to better overall performance.

## 6. Conclusions

Spectral reconstruction (SR) recovers high-resolution radiance spectra from RGB images. Many methods are regression-based, with simple formulations and usually closed-form solutions, while the current state-of-the-art spectral recovery is delivered by the much more sophisticated Deep Neural Network (DNN) solutions.

Recently, the top DNN models are trained and evaluated based on Mean Relative Absolute Error (MRAE)—a relative error which measures the spectral difference as a percentage of the ground-truth spectral value. Comparatively, all regressions are still trained based on the Least Squares (LS) minimization, which does not suggest a minimized MRAE result. This problem is further compounded by the sub-optimal regularization setting used in conventional regressions where all spectral channels are jointly regularized by a single penalty parameter.

In this paper, we developed new regression approaches that minimize relative errors and are regularized per spectral channel, including the closed-form Relative-Error Least Squares (RELS) and the Relative-Error Least Absolute Deviation (RELAD) approach (which directly minimizes MRAE and was solved by an iterative method).

Our results showed that the new minimization approaches significantly improve the conventional regressions especially in the darker regions of the images. Consequently, our best improved regression model narrows the performance gap with the leading DNNs to only 8% under the MRAE evaluation.

## Figures and Tables

**Table 1:** List of considered regression models.

Model	Abbreviation
Linear Regression [52]	LR
Root-Polynomial Regression (6th order) [56]	RPR
A+ Sparse Coding [55]	A+
Radial Basis Function Network [54]	RBFN
Polynomial Regression (6th order) [53]	PR

**Table 2:** List of minimization approaches.

Approach	Abbreviation	Per-Channel Regularization	Loss Metric
Least Squares	LS	✗	squared RMSE
Per-Channel Least Squares	LS${}^{\mathrm{pc}}$	✓	squared RMSE
Relative Error Least Squares	RELS	✓	squared relative-RMSE
Relative Error Least Absolute Deviation	RELAD	✓	MRAE

**Table 3:** The mean per-image-mean and per-image-99-percentile MRAE for all tested spectral reconstruction models. The result of HSCNN-R (the reference DNN model) is given in the last row. For all regression approaches (all except HSCNN-R), best results among the 4 training criteria—LS, LS${}^{\mathrm{pc}}$, RELS, and RELAD—are underlined.

	Mean Per-Image-Mean MRAE				Mean Per-Image-99-Percentile MRAE			
	LS	LS${}^{\mathrm{pc}}$	RELS	RELAD	LS	LS${}^{\mathrm{pc}}$	RELS	RELAD
LR	0.0624	0.0605	0.0563	0.0536	0.1695	0.1779	0.1409	0.1656
RPR	0.0469	0.0431	0.0418	0.0471	0.1548	0.1498	0.1294	0.1432
A+	0.0387	0.0375	0.0360	0.0349	0.1526	0.1428	0.1350	0.1402
RBFN	0.0206	0.0203	0.0198	0.0203	0.0789	0.0793	0.0740	0.0799
PR	0.0195	0.0193	0.0188	0.0192	0.0710	0.0709	0.0703	0.0734
HSCNN-R	0.0173				0.0653			

**Table 4:** The paired two-sample Student’s *t*-test scores of the mean per-image-mean MRAE results. For each regression model, the best minimization criterion (the one has the lowest number in Table 3) is tested against each of the other criteria.

		Student’s *t*-Test Score of Mean Per-Image-Mean MRAE			
	Best Approach	Best vs. LS	Best vs. LS${}^{\mathrm{pc}}$	Best vs. RELS	Best vs. RELAD
LR	RELAD	9.46	9.54	4.18	N/A
RPR	RELS	7.70	2.65	N/A	13.28
A+	RELAD	6.93	7.19	7.69	N/A
RBFN	RELS	4.42	3.01	N/A	5.33
PR	RELS	4.59	4.18	N/A	4.33

**Table 5:** The training and reconstruction time measurements. The size of the reconstructed images (from the ICVL database) is 1392×1300 with 31 spectral channels, and the training set (for each cross-validation trial) is composed of 100 images.

	Training Time				Reconstruction Time			
	(Per Cross-Validation Trial)				(Per Image)			
	LS	LS${}^{\mathrm{pc}}$	RELS	RELAD	LS	LS${}^{\mathrm{pc}}$	RELS	RELAD
LR	6.7 min	6.1 min	6.2 min	36.0 h	3.3 s	3.3 s	3.3 s	3.3 s
RPR	17.0 min	16.9 min	35.9 min	47.8 h	10.8 s	10.8 s	10.8 s	10.9 s
A+	26.9 min	52.8 min	54.6 min	46.2 h	1.5 min	1.5 min	1.5 min	1.5 min
RBFN	1.0 h	1.0 h	1.2 h	36.8 h	7.0 s	7.0 s	7.0 s	7.0 s
PR	15.1 min	15.4 min	42.2 min	44.6 h	9.8 s	10.0 s	10.0 s	9.8 s
HSCNN-R	35.5 h (GPU-accelerated)				3.4 min			

## Data Availability

Publicly available datasets were analyzed in this study. This data can be found here: http://icvl.cs.bgu.ac.il/hyperspectral/.

## Supplementary Materials

The code of the methods introduced in this paper is available at https://github.com/EthanLinYitun/On_the_Optimization_of_Regression_Based_Spectral_Reconstruction.
